# Découverte fortuite de quadruplets au cours d'un accouchement: illustration d'un cas à l'Hôpital Central de Yaoundé (Cameroun)

**DOI:** 10.11604/pamj.2014.18.196.4819

**Published:** 2014-07-05

**Authors:** Florent Ymele Fouelifack, Jeanne Hortence Fouedjio, Madye Ange Ngo Dingom, Jovanny Tsuala Fouogue, Robinson Mbu Enow

**Affiliations:** 1 Unité de Gynécologie-Obstétrique de l'Hôpital Central de Yaoundé, Cameroun; 2 Département de Gynécologie-Obstétriques de la Faculté de Médecine et des Sciences Biomédicales de l'Université de Yaoundé I, Cameroun

**Keywords:** Grossesse multiple, grossesse de haut rang, quadruplés, accouchement, Cameroun, multiple pregnancy, high risk pregnancy, quadruplets, childbirth, Cameroon

## Abstract

Les auteurs rapportent une grossesse de haut rang (quadruplés) dont le nombre de fœtus n'a été découvert que pendant l'accouchement par voie vaginale. Faute de moyens, la parturiente reçue en phase active du travail n'a pas pu bénéficier de la césarienne d'urgence indiquée pour présentation en siège du premier jumeau. Ce n'est qu'après l'accouchement du deuxième fœtus que les deux derniers quadruplés ont été successivement découverts. Les difficultés et les pièges contextuels de la prise en charge des grossesses multiples sont passés en revue. Ce cas rappelle au personnel des salles d'accouchement la possibilité d'erreur de diagnostique sur les résultats d'échographies présentés par les parturientes.

## Introduction

Les grossesses multiples comportent un risque qui augmente avec le nombre de fœtus [1]. La grossesse quadruple a une fréquence de 1/500000 naissances vivantes en conception naturelle d'après la formule de Hellin-Zenleny [2]. La rareté de cette entité impose une attention particulière et une prise en charge pluridisciplinaire incluant l'obstétricien, l'échographiste et le néonatologiste. Elle se conçoit dans des structures spécialisées, ce qui n'est pas souvent le cas dans les milieux aux ressources limitées. A la veille de la clôture des objectifs du millénaire pour le développement en matière de santé génésique, l'accès au suivi prénatal de qualité reste faible au Cameroun [3]. Nous illustrons un cas de grossesse quadruple découvert au cours d'un accouchement en milieu urbain.

## Patient et observation

Il s'agit d'une ménagère de 27ans, G6P3023 rendue à 39 semaines et 2 jours de grossesse. Elle est reçue en urgence pour douleurs lombo-pelviennes à type de contractions utérines. L'histoire révèle que le début de la symptomatologie remontait à 24 heures avant la consultation par la survenue d'une douleur pelvienne. Cette douleur était à type de crampes irradiant vers la région lombaire, intermittente et de fréquence progressivement croissante. Il n'y avait ni écoulement vaginal, ni de fièvre associée.

Comme antécédents, la patiente était G6P3023. La première, la troisième et la cinquième grossesse se sont terminées par des accouchements normaux à terme de 2 garçons et d'une fille. La deuxième et la quatrième grossesse se sont terminées par des avortements spontanés complets, respectivement à 08 et 12 semaines sans complications. La sixième grossesse était en cours avec un âge gestationnel de 39 semaines et 2 jours. Le suivi de la grossesse avait été assuré par un obstétricien dans un hôpital de district. Six consultions prénatales s'étalant sur les 6 derniers mois de la grossesse avaient été réalisées et aucune pathologie n'avait été rapportée. La seule échographie obstétricale disponible avait été faite à 31 semaines et 6 jours par un radiologue. Elle concluait à une grossesse intra-utérine gémellaire di-amniotique di-choriale évolutive de 26 semaines de grossesse, inférieure au terme clinique. Les fœtus de sexes masculin et féminin avaient des poids estimés de 996 et 994 grammes. Le seul examen biologique réalisé était le test de dépistage de la syphilis dont le résultat était négatif. Les prophylaxies anti-anémique et antitétanique avaient été bien administrées, cependant elle n'avait reçu aucune prophylaxie anti palustre. La parturiente a eu les ménarches à 15 ans et le premier coït à 16 ans. Son cycle menstruel est régulier à 29 jours et ses règles durent 4 jours. Elle n'avait jamais été opérée et son groupe sanguin n'était pas connu. A l'enquête des systèmes on notait en plus du motif de consultation de légères céphalées en casque.

**Figure 1:**
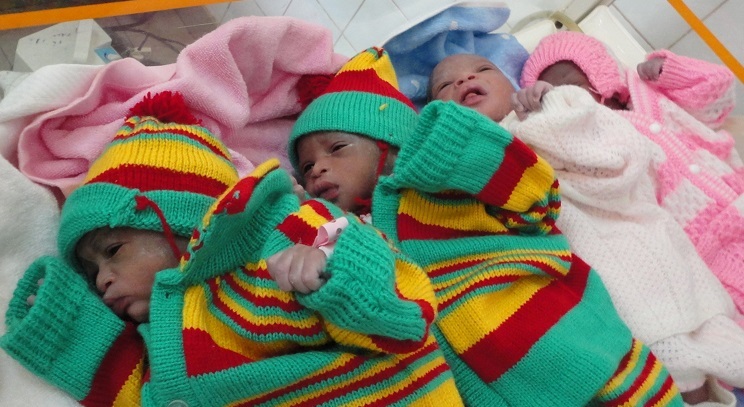
Photographie montrant les quadruplets déjà habillés

**Figure 2:**
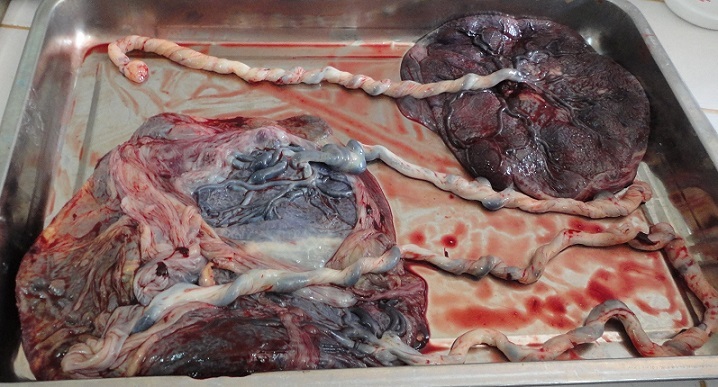
Photographie montrant les trois placentas après la délivrance

A l'examen physique, la patiente avait un bon état général. Elle était consciente et bien orientée dans le temps et dans l'espace. Elle avait une pression artérielle de 110/60 millimètres de mercure, un pouls à 88 pulsations par minute, une température de 37,3 degrés Celsius et une fréquence respiratoire de 18 cycles par minute. Les conjonctives étaient colorées et les sclérotiques anictériques. L'examen cardio-respiratoire était normal. L'abdomen était distendu avec une hauteur utérine de 51 centimètres. On y palpait quatre pôles fœtaux, orientant vers une grossesse gémellaire avec les 2 fœtus en présentation de siège. Nous avons objectivé deux foyers de bruits de cœur fœtal respectivement à 141 et 129 battements par minute. On notait 2 contractions utérines de 30 secondes chacune, toutes les 10 minutes, et de moyenne intensité. Au toucher vaginal le col était médian, effacé à 70% et dilaté à 5 centimètres. Le premier jumeau était en présentation de siège complet, les membranes intactes et il n'y avait pas de présentation du cordon. Le reste de l'examen clinique était sans particularité. Le diagnostic de travail était celui de grossesse gémellaire en phase active de travail avec le premier jumeau en siège. Une césarienne en urgence avait aussitôt été indiquée. Seulement, l'indigence de la patiente et l'absence de kits pour les urgences chirurgicales dans la ville cette nuit là n'ont pas permis de la réaliser. Après sept heures d'évolution sous surveillance par un partogramme, la parturiente est parvenue à dilatation complète avec le premier jumeau à la station +1. Nous avons décidé de procéder à l'accouchement par voie basse, au cours duquel nous avons découvert successivement des quadruplés tous en siège pesant respectivement 1730 grammes (sexe masculin, score d'Apgar 6/10 à la 1ère minute et 8/10 à la 5ème minute), 1680 grammes (sexe masculin, score d'Apgar 6/10 à la 1ère minute et 8/10 à la 5ème minute), 2130 grammes (sexe féminin, d'Apgar 8/10 à la 1ère minute et 10/10 à la 5ème minute) et 1700 grammes (sexe féminin, score d'Apgar 9/10 à la 1ère minute et 9/10 à la 5ème minute) (Figure 1). L'accouchement du quatrième fœtus s'est compliqué d'une fracture simple du fémur gauche. La délivrance active des deux placentas dont l'un tri-amniotique, était complète (Figure 2). Les nouveaux nés ont été transférés en néonatologie. La maman a été observée à la maternité pendant trois jours. La période puerpérale s'est déroulée sans complications et l'accouchée a reçu un dispositif contraceptif intra-utérin au cuivre. La fracture du fémur était complètement consolidée un mois après l'accouchement.

## Discussion

La fréquence des grossesses multiples est en nette augmentation depuis les années 1990. Aux Etats-Unis, l'incidence des accouchements multiples a atteint le pic de 193,5 pour 100000 naissances vivantes en 1998 [4]. En Europe cette incidence varie d′un pays à l′autre. La France est passée d'un taux de 9, 9 pour 100000 accouchements en 1972 à un pic de 45,7 pour 100000 accouchements en 1989 [5]. La fréquence de grossesses multiples augmente avec la présence d'antécédent familial de gémellité et la parité de la femme. Elle est environ deux fois plus fréquente chez les multipares que chez les primipares [6, 7]. L'utilisation des techniques de procréation médicalement assistée est l'un des facteurs cités dans la littérature. Notre parturiente était multipare et a conçu naturellement. La prise en charge adéquate des grossesses multiples implique une un dépistage précoce et la prévention des complications maternelles et fœtales. Le suivi de la grossesse doit être multidisciplinaire et mené par l'obstétricien, le radiologue et le néonatologiste. Les grossesses multiples sont des grossesses à haut risque et l'échographie du premier trimestre est la pierre angulaire dans leur diagnostic [8]. Notre parturiente était suivie par un obstétricien, qui a réalisé 6 consultations prénatales réparties sur les deux derniers trimestres. Le bilan prénatal biologique était incomplet. Ceci peut s'expliquer par le faible niveau socio-économique de notre patiente. Actuellement au Cameroun le coût global d'un bilan prénatal complet s'élève à 79500 francs CFA (XAF) soit environ 165 Dollars US. La réalisation de l'échographie obstétricale au cours du premier trimestre permet de déterminer le nombre de fœtus d'une grossesse et d'en étudier la chronicité [8].

Cependant cet examen est hautement dépendant du contexte clinique et de l'expérience de l'opérateur [8]. Dans notre cas l'unique échographie obstétricale disponible présentait des lacunes. Un counseling pour l'encouragement des clientes à commencer les visites prénatales précocement, un renforcement des capacités opérationnelles des échographistes en activité et la dotation des salles de travail d'appareils d'échographie pourraient à l'avenir éviter de telles situations. La connaissance à travers l'échographie du nombre de fœtus nous aurait permis de prévoir et de préparer la parturiente à une césarienne prophylactique lors des consultations prénatales pour éviter les complications liées à l'accouchement des grossesses multiples. Pour notre patiente, bien que la césarienne en urgence ait été décidée, le manque de moyens financiers et l'absence de kit d'urgence dans la ville, nous ont obligés à réaliser l'accouchement par voie basse. Une césarienne coûte en moyenne 200.000 francs CFA (414,33 dollars US) dans les hôpitaux de référence de la ville de Yaoundé, une somme inaccessible en urgence pour la majorité des citoyens dans notre pays ou le salaire minimum interprofessionnel de croissance (SMIC) est de 28.175FCFA soit 58,37 dollars US [9]. Dans notre cas la seule complication rencontrée était une fracture du fémur d'un des nouveau-nés lors de l'extraction à la naissance, ce qui est décrit dans la littérature [10].

## Conclusion

Ce cas illustre quelques obstacles à la prise en charge de grossesses multiples dans notre milieu. L'obstétricien devrait toujours avoir à l'idée la possibilité d'erreur diagnostic en échographie obstétricale et en tenir compte lors de la prise en charge.
